# Assessing the Potency of the Novel Tocolytics 2-APB, Glycyl-H-1152, and HC-067047 in Pregnant Human Myometrium

**DOI:** 10.1007/s43032-022-01000-2

**Published:** 2022-06-17

**Authors:** Md Reduanul Hossain, Jorge M. Tolosa, Roger C. Young, Roger Smith, Jonathan W. Paul

**Affiliations:** 1 School of Medicine and Public Health, College of Health, Medicine and Wellbeing, University of Newcastle, Callaghan, NSW 2308 Australia; 2 Mothers and Babies Research Center, 1 Kookaburra Circuit, New Lambton Heights, NSW 2305 Australia; 3 Hunter Medical Research Institute, 1 Kookaburra Circuit, New Lambton Heights, NSW 2305 Australia; 4 PreTeL, Inc., Chattanooga, TN USA; 5 John Hunter Hospital, New Lambton Heights, NSW 2305 Australia

**Keywords:** Novel tocolytics, Spontaneous uterine contractions, IC_50_, 2-APB, Glycyl-H-1152, HC-067047

## Abstract

The intracellular signaling pathways that regulate myometrial contractions can be targeted by drugs for tocolysis. The agents, 2-APB, glycyl-H-1152, and HC-067047, have been identified as inhibitors of uterine contractility and may have tocolytic potential. However, the contraction-blocking potency of these novel tocolytics was yet to be comprehensively assessed and compared to agents that have seen greater scrutiny, such as the phosphodiesterase inhibitors, aminophylline and rolipram, or the clinically used tocolytics, nifedipine and indomethacin. We determined the IC_50_ concentrations (inhibit 50% of baseline contractility) for 2-APB, glycyl-H-1152, HC-067047, aminophylline, rolipram, nifedipine, and indomethacin against spontaneous ex vivo contractions in pregnant human myometrium, and then compared their tocolytic potency. Myometrial strips obtained from term, not-in-labor women, were treated with cumulative concentrations of the contraction-blocking agents. Comprehensive dose–response curves were generated. The IC_50_ concentrations were 53 µM for 2-APB, 18.2 µM for glycyl-H-1152, 48 µM for HC-067047, 318.5 µM for aminophylline, 4.3 µM for rolipram, 10 nM for nifedipine, and 59.5 µM for indomethacin. A single treatment with each drug at the determined IC_50_ concentration was confirmed to reduce contraction performance (AUC) by approximately 50%. Of the three novel tocolytics examined, glycyl-H-1152 was the most potent inhibitor. However, of all the drugs examined, the overall order of contraction-blocking potency in decreasing order was nifedipine > rolipram > glycyl-H-1152 > HC-067047 > 2-APB > indomethacin > aminophylline. These data provide greater insight into the contraction-blocking properties of some novel tocolytics, with glycyl-H-1152, in particular, emerging as a potential novel tocolytic for preventing preterm birth.

**Supplementary Information:**

The online version contains supplementary material available at 10.1007/s43032-022-01000-2.

## Introduction

Preterm birth (PTB), defined as birth before 37 completed weeks of gestation, is a significant determinant of neonatal mortality, disease, and disability in surviving children [1]. Spontaneous premature labor (PTL) is the leading cause of PTB, accounting for approximately 45% of cases [1]. The risk of PTL is increased by prior preterm birth, preterm premature rupture of the membranes (PPROM), uterine overdistension, stress, and immunologically mediated processes [1, 2]. In attempts to prevent PTB, various tocolytics have been trialed for blocking spontaneous PTL, with the goal of extending pregnancy long enough for corticosteroid administration to mature the fetal lungs and improve birth outcomes. Tocolytic compounds examined include betamimetics, such as salbutamol and ritodrine, calcium ion (Ca^2+^) channel blockers (CCBs), such as nifedipine (NIF), Ca^2+^ competitors, such as magnesium sulfate, inhibitors of prostaglandin-endoperoxide synthase 2 (PTGS2), such as indomethacin (IND), oxytocin (OT) receptor antagonists (OTRA), such as Atosiban, and nitric oxide (NO) donors, such as nitroglycerine. These tocolytics are now well-characterized and work through either inhibiting pro-contraction signaling pathways, in particular by suppressing elevation of intracellular Ca^2+^ levels and blocking the availability and actions of uterotonins, or through activating pro-relaxation signaling pathways, in particular by raising intracellular cyclic adenosine monophosphate (cAMP) levels. Several systematic reviews and meta-analyses have been conducted in different parts of the world to assess the relative effectiveness of different tocolytic agents [3–8]. As a consequence, the guidelines for tocolytic management differ internationally [3, 9], and tocolytics licensed in one region of the world may or may not be licensed elsewhere. Where certain classes of tocolytics are not licensed, clinicians may utilize them off-label as second-line therapy. Approximately 75% of drugs used in obstetrics for tocolysis are unlicensed [10], this is because there are no specific therapeutic agents explicitly developed for tocolytic management except atosiban. Most tocolytics currently in use were developed for other medical indications, but were found to have tocolytic actions [10]. Moreover, there is no single agent currently available as a first-line tocolytic that is not associated with risks of side-effects [11]. Atosiban is associated with fewer side-effects than other tocolytics, but there is little evidence of efficacy [12]. All currently available tocolytic agents have relatively limited efficacy in postponing PTL and improving neonatal outcomes. As such, there remains a pressing need to evaluate the myometrial contraction blocking capabilities of novel drugs to improve tocolytic therapy.

Here, we report a comprehensive ex vivo analysis of three novel contraction-blocking agents: 2-aminoethoxydiphenyl borate (2-APB), glycyl-H-1152 dihydrochloride (GH), and HC-067047. We examined the potency of these “novel tocolytics” against spontaneous pregnant human myometrial contractions ex vivo and compared them to previously studied tocolytics, including NIF and IND, which have been used clinically for preventing PTB, as well as rolipram (ROL) and aminophylline (AMP), which have been previously investigated for uterine contraction inhibition.

2-APB was originally introduced as an inhibitor of inositol trisphosphate (IP_3_) receptors (IP_3_R) [13], which are a family of closely related Ca^2+^ channels embedded within the sarcoplasmic reticulum (SR). However, subsequent studies in intact cells have revealed that 2-APB also inhibits Ca^2+^ entry via store-operated channels (SOC); an effect that is independent of IP_3_R inhibition [14–16]. Thus, 2-APB has non-specific inhibitory effects on both IP_3_R and SOC, as well as on other Ca^2+^ transporters, e.g., sarcoplasmic Ca^2+^ ATPase (SRCA) pumps and transient receptor potential (TRP) channels of the TRPC family [17–20]. In maintaining Ca^2+^ homeostasis, G-protein coupled receptors, via activation of phospholipase C (PLC), generate intracellular IP_3_. IP_3_ then diffuses rapidly within sarcoplasm (cytoplasm) to bind with IP_3_Rs and release intracellular Ca^2+^ stores from the SR into the sarcoplasm [21–23]. The resulting increase in intracellular Ca^2+^ triggers the slow activation of SOCs in the sarcolemma (plasma membrane), which mediates store-operated Ca^2+^ entry (SOCE) into the sarcoplasm. Entry of Ca^2+^ through the sarcolemma contributes to the refilling of the SR Ca^2+^ stores and to the total intracellular pool of free Ca^2+^ in the sarcoplasm. Sarcoplasmic Ca^2+^ then binds to calmodulin, which activates the canonical signal transduction pathway that culminates in myosin light chain (MLC) phosphorylation and the actin-myosin cross bridge cycling that generates contractility [24, 25]. By inhibiting the activity of both SOC and IP_3_Rs, as well as other Ca^2+^ transporters, 2-APB prevents the elevation of sarcoplasmic Ca^2+^ levels, and in turn, inhibits myometrial contractility. Since its discovery [13], 2-APB has been tested on myometrial strips of rodents in several studies where it was found to inhibit both agonist-stimulated (OT, pennogenin tetraglycoside, *Lannea acida* plant extract, Ficus deltoidea plant extract) and spontaneous myometrial contractions [26–32]. However, despite potently inhibiting pregnant rodent uterine contractility, the effects of 2-APB on spontaneous pregnant human myometrial contractions were yet to be examined.

GH is a selective inhibitor of rho-kinase (ROCK). ROCK increases the sensitivity of uterine myocytes to Ca^2+^ through increased phosphorylation of MLC [33]. ROCK expression (mRNA abundance and protein levels) is upregulated at the end of pregnancy and is likely involved in the processes that underpin the increased myometrial contractility at term [34]. Thus, by inhibiting ROCK, GH reduces the sensitivity of uterine myocytes to Ca^2+^. GH has been found to inhibit both spontaneous and OT-stimulated pregnant human myometrial contractions ex vivo [35, 36]; however, comprehensive dose–response analyses were yet to be conducted to determine the potency of GH compared to other tocolytics.

HC-067047 is an inhibitor of transient receptor potential subfamily V, member 4 (TRPV4), a nonselective cation channel that is permeable to extracellular Ca^2+^ [37–40]. TRPV4 is activated by physiological stimuli, including stretch, swelling, heat, and pressure that may be relevant to human labor [41, 42]. Through inhibiting TRPV4 channels, HC-067047 prevents the influx of extracellular Ca^2+^ through these channels, thus preventing elevation of intracellular Ca^2+^ levels and myometrial contractility. *TRPV4* is highly expressed in pregnant human myometrium and TRPV4 protein levels increase as gestation progresses [43]. These findings suggest that TRPV4 inhibition could be a potential novel tocolytic strategy [43, 44]; however, the effects of TRPV4 inhibitors, such as HC-067047, on spontaneous pregnant human myometrial contractions were yet to be investigated.

We also examined the non-selective phosphodiesterase (PDE) inhibitor, AMP, and the selective PDE4 inhibitor, ROL. By inhibiting PDEs, which are responsible for the breakdown of cAMP, both AMP and ROL raise intracellular cAMP levels, which promotes uterine relaxation. The tocolytic effects of theophylline, the active ingredient of AMP, and ROL have been reported in pregnant rodent and human myometrium [45–51]. However, their potency in suppressing spontaneous pregnant human myometrial contractions was yet to be comprehensively assessed. We also determined the potency of NIF and IND, which have well-documented tocolytic effects.

**Fig. 1:**
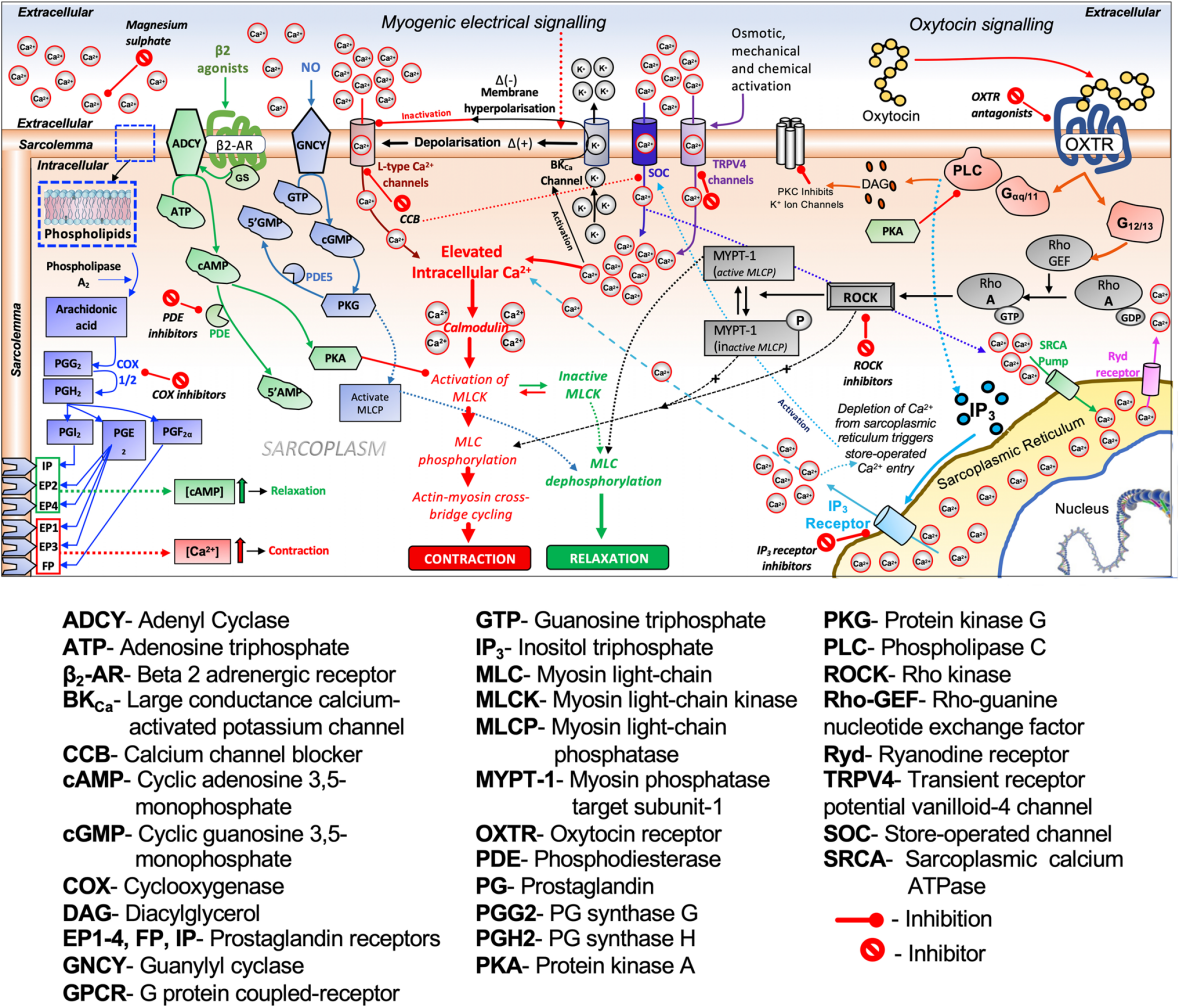
Overview of myometrial contraction signaling pathways and tocolytic action. The tocolytic agents examined block myometrial contractility through either inhibiting pro-contraction signaling pathways (2-APB, glycyl-H-1152, HC-067047, nifedipine, and indomethacin) or through activating pro-relaxation signaling pathways (aminophylline, rolipram)

We conducted comprehensive dose–response analyses for 2-APB, GH, HC-067047, AMP, ROL, NIF, and IND using strips of pregnant human myometrium undergoing spontaneous contractions ex vivo. The location at which each of these agents affects uterine myocyte contraction signaling pathways is shown in Fig. 1. We then compared the contraction-blocking potency of the agents to assess the tocolytic potential of 2-APB, GH, and HC-067047 as novel tocolytics.

## Materials and Methods

### Drugs and Reagents

Drugs and reagents were obtained from the following sources: NIF (cat No N7634), IND (cat No I7378), and AMP (cat No A1755, MW-210.21) were purchased from Sigma-Aldrich Pty Ltd (Sydney, Australia); ROL (cat No 0905), GH (cat No 2485, batch-specific MW 458.3), 2-APB (cat No 1224), and HC-067047 (cat No 4100) were purchased from Tocris (Bristol, UK). Other miscellaneous reagents were purchased from Sigma-Aldrich Pty Ltd (Sydney, Australia) and ThermoFisher Scientific Inc (Sydney, Australia).

### Human Myometrial Specimens

Biopsy specimens of human myometrial tissue were obtained from women, undergoing elective cesarean section at the John Hunter Hospital, NSW, Australia. Biopsies were collected with the approval of the Hunter and New England Area Human Research Ethics Committees (2019/ETH12330) and all participants gave informed written consent. Myometrial biopsies were obtained from the upper lip of the incision in the lower uterine segment. All myometrial biopsies were obtained from term pregnancies (37–40 weeks of gestation) where the woman was not-in-labor (NIL). The clinical indications for elective cesarean section were breech presentation or a previous cesarean. All women were examined clinically and those with signs of infection, or with diabetes mellitus, or treated with any medication other than prenatal vitamins were excluded from the study. Patient demographic data are shown in Table 1. Upon collection, the myometrial biopsies were placed in pre-chilled phosphate buffered saline (PBS) on ice for transportation and were used within 60 min to commence myometrial dose–response contraction assays.

**Table 1:** Demographic characteristics of the pregnant women

Characteristics	Value
Maternal age (Y)^a^	33.3 ± 4.5
Maternal body mass index (kg/m^2^)^a^	26.9 ± 4.8
Gestational age (weeks)^a^	38.8 ± 0.8
Fetal body weight during birth (g)^a^	3616 ± 436
Gravida (n%)	
*1*	3 (15)
*2*	11 (55)
*3*	5 (25)
*4*	1 (5)
Parity (n%)	
*0*	3 (15)
*1*	14 (70)
*2*	3 (15)
Indication of elective cesarean delivery (n%)	
*Previous cesarean delivery*	15 (75)
*Breech*	5 (25)

^*a*^*Data are shown as mean* ± *standard deviation. Maternal age and gestational age are shown as the age during cesarean delivery. The body mass index was recorded at 1st visit/pregnancy booking (usually at 12–28 weeks of gestation)*

### Myometrial Contraction Assays

Myometrial contraction assays were performed as previously described [52–54] using an 8-channel Radnoti Tissue-Organ Bath System (Radnoti Glass Technology Inc., Monrovia, CA, USA) equipped with MLT0201 force transducers (ADInstruments, Bella Vista, NSW, Australia) and eight temperature-controlled organ baths. Human myometrial specimens were dissected into 8 × 1.5 × 1.5 mm tissue strips then connected to the force transducers using nylon thread and stainless-steel tissue clips (ADInstruments). Each strip was lowered into a separate organ bath containing 15 mL modified Krebs–Henseleit buffer solution (KREBS) (no Ca^2+^, no NaHCO_3_) (Sigma-Aldrich, cat no K3753-10X1L) supplemented to 2.5 mM CaCl_2_ and 25 mM NaHCO_3_. Organ baths were maintained at 37 °C and KREBS continuously gassed with 95% O_2_ and 5% CO_2_. The transducer position was adjusted to apply 1 g of tension to each strip. The strips were then equilibrated whereby every 10 min for a total of 30 min, the organ baths were drained then refilled with 15 mL of KREBS (tissue strips washed). Due to tissue creep during stabilization, tissue length was increased to return tension to 1 g. Washing and re-tensioning to 1 g was repeated twice more (each strip tensioned to 1 g a total of 3 times). Thereafter, tension stabilized between 0.5 and 0.9 g and strips were left to develop spontaneous rhythmic contractions ex vivo. Under the described conditions, the myometrial strips took approximately 2 h to establish spontaneous contractions with consistent amplitude and frequency. Testing protocols were then begun, and strips were then maintained under isometric conditions for the remainder of the experimental run. In control experiments, tension was maintained near baseline tension for 6–7 h (Fig. S1). In experiments involving serial drug additions, some treatments resulted in changes of baseline tension. To accommodate these baseline changes, AUC were calculated from the tension between contractions observed immediately prior to drug addition. Contraction data were captured and visualized in real-time using a PowerLab 8/35 data-acquisition system and LabChart software (ADInstruments). Contraction traces were analyzed for key contraction parameters, including amplitude (g) and frequency (contractions/h), and integration of these values to determine the area under the curve (AUC) (g tension × sec). AUC was considered an index of contraction performance and was calculated based on the total area for all contractions generated during each 30 min treatment window (Fig. S2).

### Longevity of Spontaneous Contractions in Pregnant Human Myometrium *Ex Vivo*

To ensure that myometrial strips were able to contract for the duration of the tocolytic studies, strips were allowed to generate spontaneous contractions ex vivo for 7 h. Traces were then analyzed (in 60 min blocks) to confirm that there was no significant change in the resting tension, contraction amplitude, contraction frequency, or AUC across the 7 h period of spontaneous ex vivo contractions.

### Dose–Response Study

Before administering drug treatments, a contraction baseline was established for each tissue strip during which 1 h of contractions of consistent amplitude and frequency was recorded. Following the establishment of the baseline, treatments were added to the organ baths and the effects on contractility recorded. For each tissue strip, cumulative concentrations of drugs were administered at 30 min intervals. The effect of each drug was assessed against each strip’s contraction baseline (each strip has an internal control). Seven drugs (2-APB, GH, AMP, HC-067047, ROL, NIF, IND) were analyzed against myometrium from different term pregnant NIL women (replicate numbers as indicated). To control for any effects of the drug vehicles (dimethyl sulfoxide (DMSO), Milli-Q water or KREBS buffer), equivalent cumulative volumes of vehicles were assessed against separate tissue strips during each contraction assay.

### Data Analysis

Analysis of AUC was performed using LabChart 8.0 Pro with the dose–response module (ADInstruments). For each strip, the last 30 min of contractions immediately prior to commencing treatments was used as the baseline (100%). Effects of treatments were normalized against the baseline and data expressed as percent (%) of baseline contractility. Dose–response curves for AUC were generated using the non-linear regression model of GraphPad Prism 8.0 (GraphPad Software Inc., San Diego, CA, USA) and fitted through the (log inhibitor vs normalized response-variable slope) equation, *Y* = 100/(1 + 10^((Log IC_50_-X)*HillSlope)). The concentration of each drug required to inhibit ex vivo myometrial contractility by 50% (IC_50_) was determined as being a 50% reduction in total AUC relative to the contraction baseline. An ordinary one-way ANOVA followed by Dunnett’s multiple comparisons test was used to determine significant differences between the baseline and mean AUC of each dose used in the dose–response curve. A probability (P) value of < 0.05 was considered statistically significant.

### Confirmation of IC_50_

We sought to confirm the accuracy of the IC_50_ values determined for each drug. Baseline contractility for tissue strips was recorded for 1 h. Each drug was then applied to individual contracting strips as a single treatment at the IC_50_ determined for each drug (with exception of HC-067047, which could not be solubilized at the required concentration predicted to be the IC_50_). Contractility was recorded for a further 1 h. The effect of administering each drug at the IC_50_ on AUC was then determined.

## Results

### Longevity of Spontaneous Contractions and Assessment of Drug Vehicles

We first sought to confirm that term NIL myometrial strips were able to maintain consistent spontaneous rhythmic contractions ex vivo for 7 h, which was the maximum duration of cumulative tocolytic treatments. The strips exhibited spontaneous rhythmic contractions within 2 h of the final equilibration wash/re-tension (Fig. S2, panel A). Contractions remained stable for over 7 h in that comparison of the 60 min periods revealed no significant changes in resting tension, contraction amplitude, frequency, or AUC (*n* = 5) across the 7 h period (Fig. S2, panels B–E).

During the treatment time courses (2.5–3.5 h), the administration of cumulative doses of DMSO (maximum of 0.42%), Milli-Q water, or KREBS (drug vehicles) had no effect on the contraction amplitude, frequency, or AUC (Fig. S3, panel A–C and Fig. 3, panel A–G).

### Dose–Response Analyses

**Fig. 2:**
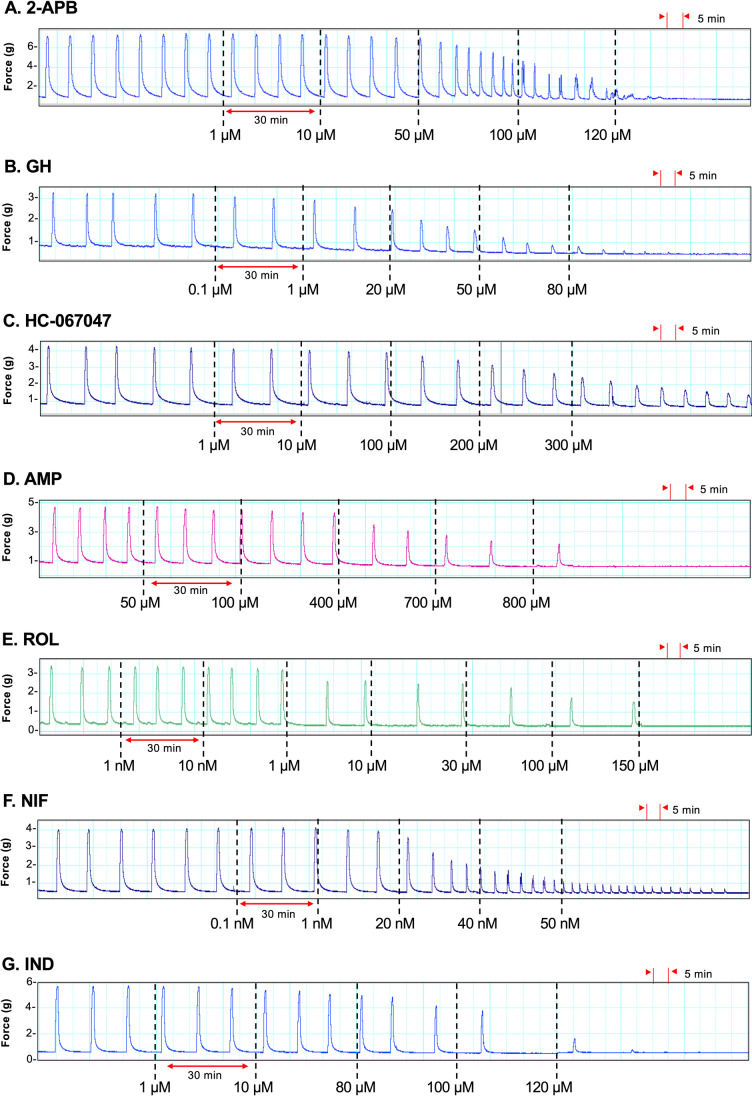
Representative traces showing the effect of cumulative drug treatments on contractions. Spontaneously contracting pregnant human myometrial strips were treated with cumulative concentrations of **A** 2-APB (*n* = 8), **B** GH (*n* = 8), **C** HC-067047 (*n* = 7), **D** AMP (*n* = 8), **E** ROL (*n* = 8), **F** NIF (*n* = 10), and **G** IND (*n* = 10). Dotted lines indicate the points at which the treatment was added to the bath

Each of the drugs analyzed dose-dependently inhibited contractions in spontaneously contracting strips of pregnant human myometrium ex vivo. The appropriate dosing regimens for each drug were optimized by prior organ bath contraction studies to determine a dose–response regimen for each drug, whereby the lowest drug concentration had no significant effect, and the highest concentration exerted the maximal inhibitory effect on contractions (IC_max_). For all drugs except HC-067047, IC_max_ was the abolition of contractions (0% of baseline AUC) (data not shown). All drugs, except HC-067047, were therefore equally effective at abolishing spontaneous pregnant human myometrial contractions ex vivo but exhibited different potencies (different IC_50_ values) (Fig. 2).

The cumulative concentrations of the novel tocolytics, 2-APB (1–120 µM), GH (0.1–80 µM), and HC-067047 (1–300 µM), affected spontaneous contractions similarly in that for each of these drugs, as contraction amplitude was reduced, contraction frequency significantly increased (Fig. 2, panels A–C). In contrast, as AMP (50–800 µM) and ROL (0.001–150 µM) inhibited contraction amplitude, contraction frequency significantly decreased (Fig. 2, panels D–E). This was particularly the case for ROL. The effects of NIF (0.1–50 nM) were consistent with the effects of 2-APB, GH, and HC-067047, in that as NIF reduced contraction amplitude, contraction frequency increased (Fig. 2, panel F), while the effects of IND (1–120 µM) were consistent with AMP and ROL, in that IND reduced contraction frequency as amplitude was inhibited (Fig. 2, panel G).

### Tocolytic IC_max_

2-APB and GH abolished spontaneous ex vivo contractions at 120 and 80 µM, respectively, whereas HC-067047 failed to completely abolish contractions even at the highest cumulative concentration tested (300 µM). At concentrations of ≥ 100 µM, HC-067047 precipitated out of solution within the organ baths. As such, the contractility recorded at the 100, 200, and 300 µM concentrations does not accurately reflect the effects of HC-067047 against spontaneous pregnant human myometrial contractions ex vivo. AMP and ROL abolished spontaneous contractions (IC_max_) at 800 and 150 µM, respectively. The traditional tocolytics, NIF and IND, abolished contractions at 50 nM and 120 µM, respectively.

### Determination of the Half-Maximal Inhibitory Concentration (IC_50_)

**Fig. 3:**
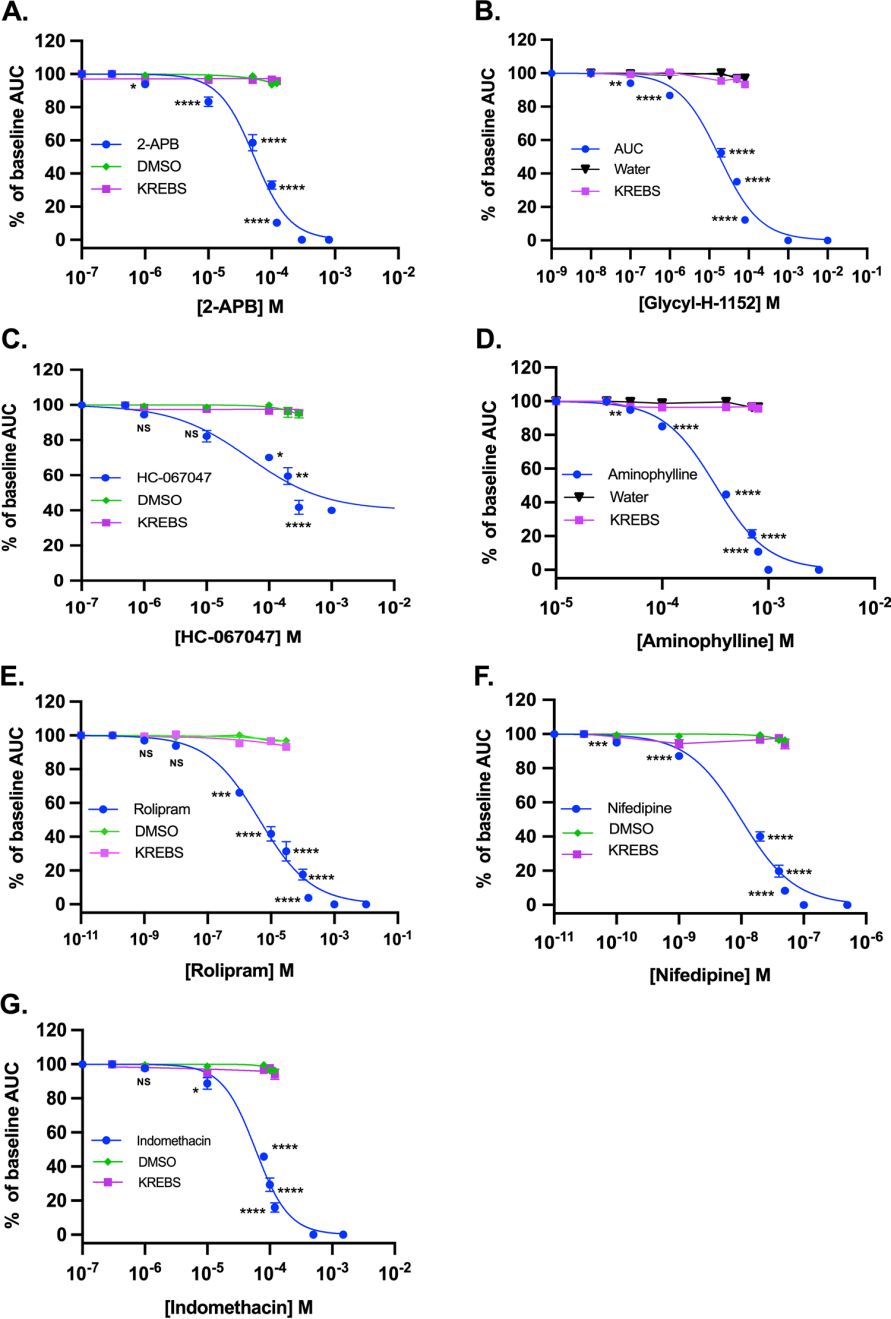
Dose–response curves. Plotted dose–response curves for the effect of **A** 2-APB (*n* = 8), **B** GH (*n* = 8), **C** HC-067047 (*n* = 7), **D** AMP (*n* = 8), **E** ROL (*n* = 8), **F** NIF (*n* = 10), and **G** IND (*n* = 10) on spontaneous pregnant human myometrial contractions ex vivo. Contractility was measured as AUC and expressed relative to the contraction baseline. Data are presented as mean ± SEM. There was a significant reduction in AUC in response to the cumulative doses of drugs. Comparisons were made between baseline and mean AUC of each dose using ordinary one-way ANOVA followed by Dunnett’s multiple comparisons test. A probability (P) value of < 0.05 was considered statistically significant. The *asterisks* indicate a significant difference, where 4 *asterisks* indicate *P* < 0.0001

To determine tocolytic potency, dose–response curves were generated (Fig. 3, panels A–G) then the IC_50_ value determined for each drug. For the novel tocolytics, IC_50_ values were determined to be 53 µM for 2-APB (*n* = 8), 18.2 µM for GH (*n* = 8), and 48 µM for HC-067047 (*n* = 7). For the PDE inhibitors, IC_50_ values were 318.5 µM for AMP (*n* = 8) and 4.3 µM for ROL (*n* = 8), while for the traditional tocolytics, IC_50_ values were 10 nM for NIF (*n* = 10) and 59.5 µM for IND (*n* = 10). Contractility data are summarized in Table 2.

**Table 2:** Summary of experimentally determined IC_50_ and IC_max_ concentrations for each tocolytic

Drug name			AUC based parameters		
	**IC**_**50**_	**9B**		**Hill slop**e	**IC**_**max**_
2-APB	53 μM	(42.7–63.5) μM		1.5	120 μM
Glycyl-H-1152	18.2 μM	(14.4–22.7) μM		0.84	80 μM
HC-067047	48 μM	(38.3–61.2) μM		0.71	300 μM (average of 60% inhibition of total contractility)
Aminophylline	318.5 μM	(290–350) μM		1.76	800 μM
Rolipram	4.3 μM	(3–5.8) μM		0.53	100 μM
Nifedipine	10 nM	(7.5–14.9) nM		1	50 nM
Indomethacin	59.5 μM	(47.8–83) μM		1.5	120 μM

*IC*_*50*_, *half-maximal inhibitory concentration*; *IC*_*max*_, *maximum inhibitory concentration required for complete contraction abolishment*; *Hill slope*, *slope of the dose–response curve*

### Confirmation of Half-Maximal Inhibitory Concentration (IC_50_*)*

**Fig. 4:**
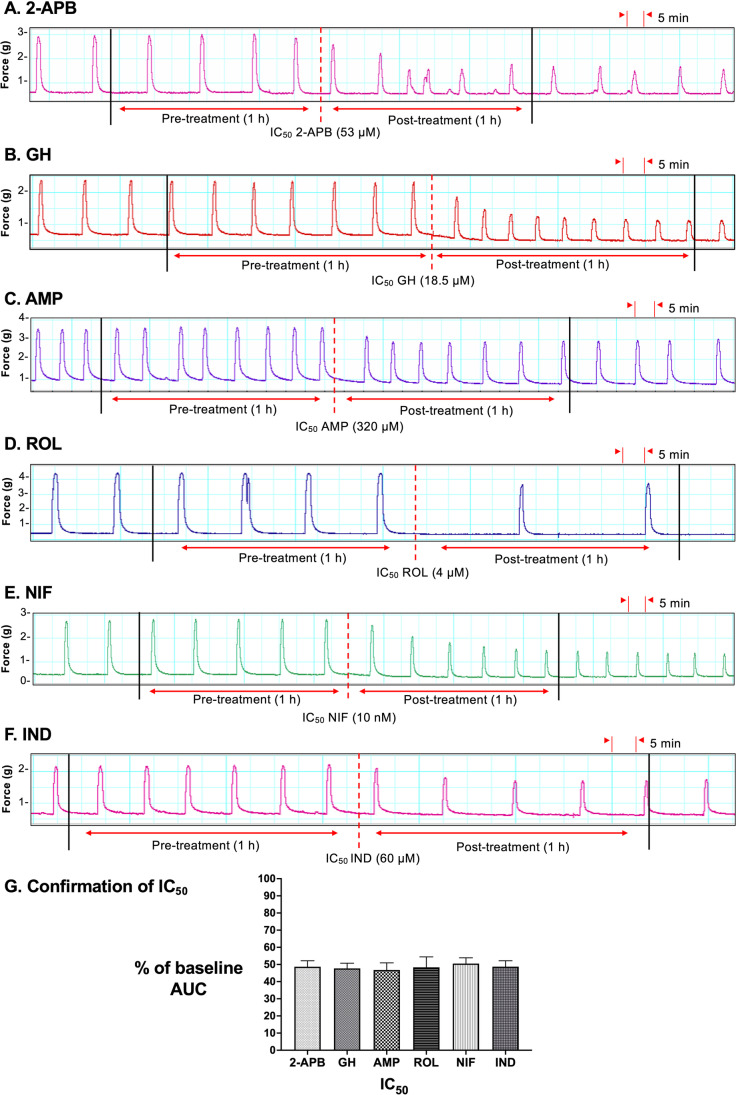
Confirmation of the experimentally determined IC_50_ values. Representative traces showing the extent of contraction inhibition following treatment of pregnant human myometrial strips with the experimentally determined IC_50_ concentrations for **A** 2-APB (*n* = 10), **B** GH (*n* = 10), **C** AMP (*n* = 10), **D** ROL (*n* = 5), **E** NIF (*n* = 10), and **F** IND (*n* = 10). Black solid lines indicate the boundaries of the 1 h pre- and post-treatment analysis windows, and red dotted lines indicate the points at which the treatment was added to the bath. **G** The mean percent inhibition (relative to the contraction baselines) induced by each drug when applied at the IC_50_ for 60 min

Having determined the IC_50_ for each drug via the dose–response analyses, we then applied the IC_50_ to contracting strips as a single treatment to ascertain the accuracy of the determined IC_50_ values. Representative contraction traces are shown in Fig. 4, panels A–F. AUC analysis (1 h pre-treatment vs 1 h post-treatment) confirmed that for each drug, AUC was reduced by approximately 50% (2-APB = 49 ± 3% (*n* = 10); GH = 48 ± 3% (*n* = 10); AMP = 46 ± 4% (*n* = 10); ROL = 48 ± 5% (*n* = 5); NIF = 50 ± 3% (*n* = 10); IND = 49 ± 3% (*n* = 10) (Fig. 4, panel G).

### The Tocolytic Effect of the Drugs Is Reversible

**Fig. 5:**
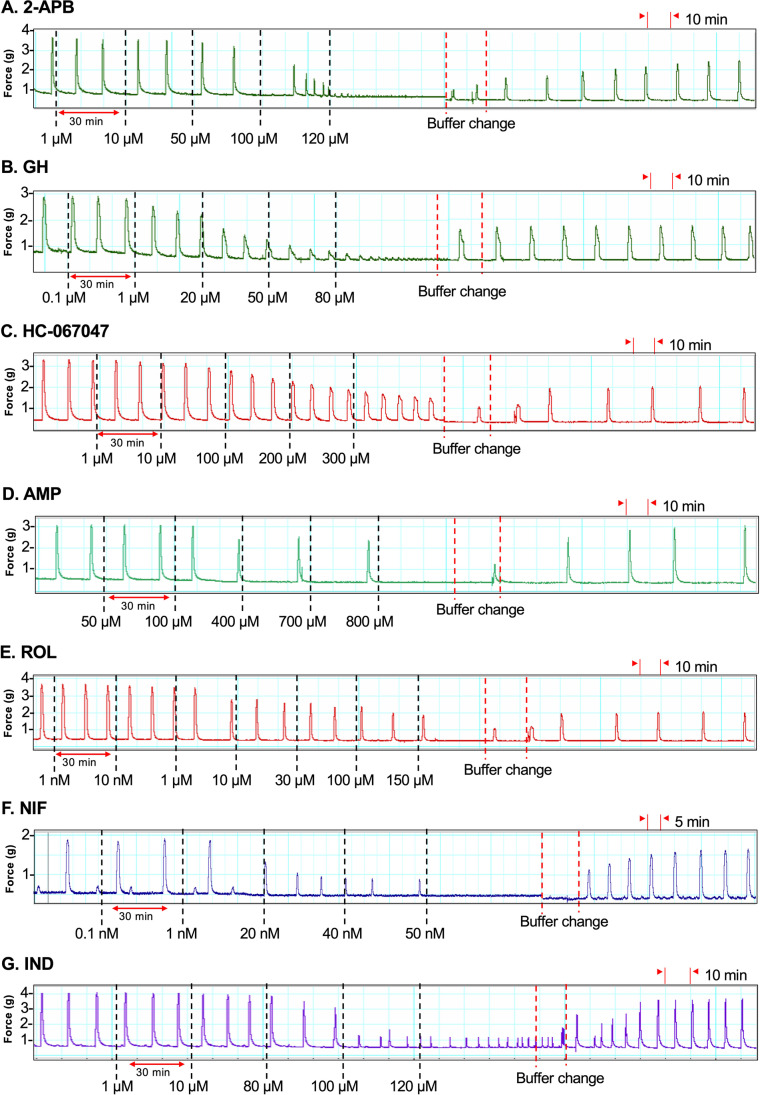
Reversibility of the tocolytics. Representative traces showing that after washout of the drugs, contractions spontaneously resumed in pregnant human myometrial strips treated with **A** 2-APB (*n* = 5), **B** GH (*n* = 5), **C** HC-067047 (*n* = 5), **D** AMP (*n* = 5), **E** ROL (*n* = 5), **F** NIF (*n* = 5), and **G** IND (*n* = 5). Red dotted lines indicate the points at which organ baths were drained then refilled with fresh KREBS buffer

To validate that the contraction inhibition was mediated by drugs and not due to diminished cellular viability and/or metabolic restriction of the tissue, the myometrial strips were washed with KREBS solution after tocolytic treatment to ascertain whether spontaneous contractions resumed. For all drugs analyzed, the strips resumed contracting after the washout procedure following a brief (< 30 min) recovery period (Fig. 5, panels A–G).

## Discussion

Evidence suggests that PTL is a syndrome attributable to multiple pathological processes, including infection or inflammation, uterine overdistension, stress, ischemia or hemorrhage, endocrine disorders, immunologically mediated processes [55], and a gene expression pattern distinct from term labor [56]. Thus, PTL is a heterogeneous condition of multiple dysfunctions of preterm tissue that may lead to myometrial contractions, membrane/decidual activation, and/or cervical ripening. These three attributes constitute the common terminal pathway of both preterm and term birth. Hence, it is unclear whether spontaneous PTL results from premature activation of the term labor process, or due to pathological insults initiating uterine transformation from quiescence to overt labor [57]. Although evidence indicates that preterm and term labor are distinct processes [56], the preterm and term labor ultimately converge at the level of the myometrial contractile proteins. In this regard, there are no data available reporting that the levels of contractile proteins relevant to this study (IP_3_R, TRPV4, L-type Ca^2+^ channels, and ROCK) change between preterm and term human myometrium. Therefore, in the absence of such data, tocolytic agents that target these proteins should not be dismissed, as they may yet have relevance to tocolysis during PTL.

In this study, we performed comprehensive dose–response analyses to examine the contraction-blocking potency of three potential new tocolytics, 2-APB, GH, and HC-067047. We compared the IC_50_ of these new tocolytics to the PDE inhibitors, AMP and ROL, and to the clinically deployed tocolytics, NIF and IND. In terms of inhibiting spontaneous pregnant human myometrial contraction ex vivo (measured as AUC), the order of potency of the tocolytics from highest to lowest was NIF > ROL > GH > HC-067047 > 2-APB > IND > AMP (Table 3).

**Table 3:** Ranking of tocolytic agents according to their contraction blocking potency

Potency ranking	All agents examined	Novel tocolytics	Previously examined tocolytics	Clinically used tocolytics
1	Nifedipine	Glycyl-H-1152	Rolipram	Nifedipine
2	Rolipram	HC-067047	Aminophylline	Indomethacin
3	Glycyl-H-1152	2-APB		
4	HC-067047			
5	2-APB			
6	Indomethacin			
7	Aminophylline			

As a non-specific inhibitor of IP_3_Rs and SOC, 2-APB blocks the Ca^2+^ entry from both intracellular stores [13] and extracellular space [14–16], which prevents the elevation of cytosolic Ca^2+^ levels that drives contractions. Suppression of myometrial contractions in vitro by 2-APB was first reported by Ascher-Landsberg et al., who showed inhibition of both OT-stimulated and spontaneous contractions in rat myometrium, with contractions ultimately abolished at 100 µM [26]. This generally aligns with other subsequent studies in rodents, where 2-APB inhibited uterine contractions stimulated by different agonists, including OT and pennogenin tetraglycoside [26–32]. In our study, 2-APB abolished spontaneous pregnant human myometrial contractions at 120 µM, which is largely consistent with Ascher-Landsberg et al. [26], and we identified an IC_50_ (50% reduction in baseline AUC) for 2-APB of 53 µM. However, unlike Gravina et al., who reported a reduction in mouse myometrial contraction frequency following treatment [29], we observed that in pregnant human myometrium, contraction frequency increased as amplitude decreased in response to cumulative 2-APB treatments. Moreover, existing literature indicates that 2-APB exerts concentration-dependent biphasic effects on both SOC and IP_3_Rs. Patch-clamp studies on intact cell lines have shown that 2-APB has a stimulatory effect on Ca^2+^ entry via SOC and the IP_3_R gating system at lower concentrations (< 10 µM), and thus caused a transient increase in the amplitude of Ca^2+^ rise, whereas higher concentrations (> 10 µM) of 2-APB inhibits Ca^2+^ entry [15, 58, 59]. However, a biphasic effect of 2-APB was not observed in our myometrial tissue strip studies. We generated concentration–response curves based on assessment of both AUC (Fig. 3) and amplitude alone (data not shown) and in both cases, we detected no stimulatory effect of 2-APB on myometrial contractility at low concentrations (1 µM). The inconsistency may be attributable to the different experimental models in that the biphasic effect of 2-APB was observed against a non-uterine myocyte cell in monoculture using patch-clamp analysis, whereas our study utilized strips of pregnant human myometrium in a contraction bioassay system [15, 58, 59]. In support of this, existing literature demonstrates that isoform expression of STIM (1–2), a SR membrane protein that induces the opening of the SOCs, and ORAI (1–3), a plasma membrane protein that forms the pore of SOCs, differs between myometrial cells in culture and myometrial tissue [60–62]. 2-APB is shown to have differential effects on different STIM and ORAI isoforms [63, 64], which together mediate SOCE. Thus, the absence of a biphasic effect in the present study may be attributable to differences in expression of STIM and/or ORAI isoforms, compared to cell lines.

By inhibiting the isoenzyme, ROCK [65], GH reduces the Ca^2+^ sensitivity of uterine myocytes. The myometrial contraction and relaxation cycle depends on the equilibrium between phosphorylation and dephosphorylation of MLC, where myosin light chain kinase (MLCK) phosphorylates MLC to promote contraction and myosin light chain phosphatase (MLCP) dephosphorylates MLC to promote relaxation. MLCK is a Ca^2+^-dependent enzyme that is activated by the formation of the Ca^2+^-calmodulin complex in response to an intracellular Ca^2+^ surge [66–69]. In contrast, MLCP is negatively regulated by a Ca^2+^-independent mechanism where a regulatory subunit of MLCP is phosphorylated by ROCK, blocking the action MLCP and potentiating the effect of MLCK. Thus, the mechanism requires less Ca^2+^ to regulate MLC phosphorylation; a phenomenon called Ca^2+^ sensitization [33]. It has recently been demonstrated that ROCK-mediated Ca^2+^ sensitization of contractility can be induced by muscarinic or OT receptor stimulation in rat and human myometrium [70]. Hudson et al. and Aguilar et al. reported that cumulative concentrations and a single concentration (1 µM) of GH inhibited both OT-stimulated and spontaneous contraction of human myometrium ex vivo [35, 36]. Our comprehensive dose–response analyses add to these data as we have shown that in spontaneously contracting pregnant human myometrium ex vivo, the IC_50_ for GH is 18.2 µM and contractions are abolished at 80 µM. Our analyses also demonstrate that as GH inhibits contraction amplitude, contraction frequency increases, similar to 2-APB.

The third novel tocolytic we examined was HC-067047, a selective inhibitor of TRPV4 channels. TRPV4 plays a role in extracellular Ca^2+^ influx in response to various stimuli (stretch, swelling, heat, or pressure). There are limited studies examining the tocolytic effect of HC-067047. In mouse and rat uterine tissue strips, a single treatment with 1 µM HC-067047 inhibited contractions stimulated by OT and GSK1016790A [43, 71]. However, when used to treat pregnant human myometrium at the same concentration (1 µM), HC-067047 had only a slight inhibitory effect against OT-stimulated contractions [72].

Interestingly, Villegas et al. reported that activation of TRPV4 channels by the TRPV4 agonists, GSK1016790A and 4αPDD, resulted in the inhibition of OT-stimulated contractions in pregnant human myometrium [73]. This was attributed to an indirect effect whereby Ca^2+^ influx through TRPV4 channels causes K^+^ efflux via BK_Ca_-channel activation, which, in turn, causes membrane hyperpolarization that inhibits L-type Ca^2+^ channels. This contrasts with the findings of Ying et al., who reported that GSK1016790A increased the contractility of mouse uterine tissue, which was then inhibited by HC-067047 [43]. Ying et al. also reported that HC-067047 inhibited OT-stimulated mouse uterine contractions, as well as delayed parturition in both RU486- and inflammation-induced mouse models of PTL [43]. Moreover, Singh et al. reported that HC-067047 inhibited GSK1016790A-induced contractility in murine strips ex vivo [71], while another ex vivo study with rat myometrial strips reported that the TRPV4 antagonist, RN1734, significantly decreased uterine contractility, whereas the TRPV4 agonist, RN1747, increased contractility [74]. The reason for the discrepancy in relation to the effects of the TRPV4 agonism is unclear but may be related to temporal and physical Ca^2+^ compartmentalization that plays a role in fine tuning contractility [72]. Nonetheless, our findings are consistent with prior studies from the mouse and rat, in that TRPV4 antagonism by HC-067047 unequivocally inhibited ex vivo spontaneous pregnant human myometrial contractions in a concentration-dependent manner (1, 10, 100 µM). Precipitation of HC-067047 at higher concentrations (200, 300 µM) means that the potency of HC-067047 is likely higher than that determined during this study. HC-067047 may therefore be a novel avenue for tocolysis; however, an effective delivery strategy may be required that overcomes the low aqueous solubility, such as delivery via uterine-targeted nanoliposomes [53] or vaginally administered mucus penetrating nanoparticles [75].

As a non-selective PDE inhibitor, AMP leads to the intracellular accumulation of cAMP, which operates through various mechanisms to promote uterine myocyte relaxation (see Fig. 1). Prior studies have demonstrated smooth muscle and myometrial contraction inhibition by AMP in rodents [48, 49, 76] and human [51, 77–81]. In an ex vivo study with pregnant human uterine strips, Bird et al. reported that AMP (40 and 100 µM) produced concentration-dependent inhibition of OT-stimulated contractions [77]. In another study, Verli et al. reported that increasing AMP concentrations (0.01 nM–10 µM) reduced OT-stimulated human myometrial contractions by 25% of baseline contractility [51]. Leroy et al., Berg et al., and Lai et al. demonstrated that AMP inhibited spontaneous pregnant human myometrial contractions in a concentration-dependent manner, where Leroy et al. and Berg et al. determined IC_50_ values for AMP of ≤ 100 µM [78, 80] and Lai et al. reported at 263 µM [81]. In the present study, AMP also inhibited spontaneous pregnant human myometrial contractions in a concentration-dependent manner; however, a high concentration (800 µM) of AMP was required to abolish contractility. Moreover, our determined IC_50_ for AMP of 318.5 µM is higher than that reported by Leroy et al., Berg et al. (100 µM), and Lai et al. (263 µM). The reason for these differences is currently unknown.

While AMP is a non-selective PDE inhibitor, ROL selectively inhibits PDE4, which is highly expressed in pregnant human myometrium at term [82]. Verli et al. reported that increasing concentrations of ROL (0.01 nM–10 µM) inhibited OT-stimulated contractions in pregnant human myometrial strips, and at the highest concentration tested of 10 µM, the authors found that ROL inhibited 62% of baseline contractility. Leroy et al., Bardou et al., and Martinez et al. reported that ROL inhibited spontaneous pregnant human myometrial contractions in a concentration-dependent manner, with 50% inhibition of contractility observed at 100 nM, 158 nM, and 22.7 µM, respectively [45, 78, 83]. In our analyses, ROL abolished ex vivo spontaneous pregnant human myometrial contractions at 150 µM and we determined the IC_50_ to be 4.3 µM. We also noted that for both AMP and ROL treatment, as contraction amplitude decreased, contraction frequency decreased; this contrasts the effects of 2-APB, GH, and HC-067047, where we observed that contraction frequency increased as amplitude was inhibited.

As an inhibitor of voltage-gated L-type Ca^2+^ channels, NIF blocks the influx of extracellular Ca^2+^ that underpins uterine myocyte contractility [84, 85]. The effects of NIF are well established [86, 87] and it is currently used clinically for tocolysis in various countries [88]. In the present study, we determined an IC_50_ for NIF of 10 nM. This is lower than the IC_50_ values reported by Moynihan et al. [87] (55 nM) and Ballejo et al. [89] (200 nM) against spontaneously contracting pregnant human myometrium, but is largely consistent with the IC_50_ values reported by Kuc et al. [90] (8.68 nM) and Longo et al. [91] (7.4 nM) against OT-stimulated contractions in pregnant human myometrium. Our determined IC_50_ of 10 nM, and with contractions abolished at 50 nM, makes NIF orders of magnitude more potent than all the other drugs examined in this study.

Within pregnant myometrium, IND blocks the synthesis of the prostaglandins that promote uterine contractions [92] and has been widely used as a tocolytic for many years. The tocolytic effect of IND was first reported by Vane et al. [93] and has been subsequently examined many times [94–98]. Across these studies, IND was reported to inhibit myometrial contractions at different concentrations and there is no emergent consensus as to an IC_50_. In our analyses, contractions were abolished by 120 µM IND and we determined the IND IC_50_ to be 59.5 µM. During prior studies on spontaneously contracting pregnant human myometrium, Arrowsmith et al. [94] and Johnson et al. [96] reported IND IC_50_ values of 35.4 and 278 µM, respectively, placing our findings within the range of these prior studies.

### Strengths and Limitations

This study was the first to conduct a comprehensive dose–response study for novel tocolytics, 2-APB, GH, and HC-067047, to determine IC_50_ concentrations and compare their potency with clinically used tocolytics. A strength of this study was our confirmation of determined IC_50_ for each drug, where we confirmed that a ~ 50% reduction in baseline AUC was achieved (with exception of HC-067047 due to solubility limitations) when each drug was applied to contracting myometrial strips as a single treatment. These data support the accuracy of our determined IC_50_ values within our experimental setting and may provide insight into the relative tocolytic potency that could be expected from the different drugs in a clinical setting.

The study has only examined tocolytic potency against unstimulated (spontaneous) ex vivo contractions in term, NIL pregnant human myometrium. The authors acknowledge that there is increasing evidence that there are distinct differences between preterm and term labor [56], which may call into question the relevance of these data gleaned from term NIL myometrium. However, as previously mentioned, there are no data available indicating that levels of IP_3_R [99–101], TRPV4 [43], and ROCK [34, 102, 103] change between preterm and term pregnant human myometrium. Additionally, changes in expression of L-type Ca^2+^ channels [104–107], PTGS1 [108, 109], PTGS2 [109], and PDE4 [51, 110, 111] have been reported between preterm and term myometrium; however, each of these proteins was reported to exhibit higher expression in preterm myometrium than at term, suggesting that tocolytics targeting these proteins may actually have greater relevance during PTL than labor at term.

Lastly, this unstimulated model is standard technique for elucidating contraction pathways; however, further valuable insight would be garnered by examining agonist-stimulated contractions, as well as term IL myometrium and preterm NIL and IL myometrium.

### Final Remarks

This study represents a comprehensive analysis of the myometrial contraction-blocking potency of the novel tocolytics, 2-APB, glycyl-H-1152, and HC-067047, and their potency comparison against the traditional tocolytics, nifedipine and indomethacin, as well as other potential candidates, rolipram and aminophylline (Table 2). Among the novel tocolytics, glycyl-H-1152 was the most potent followed by HC-067047 and 2-APB. Glycyl-H-1152 was also found to be a more potent inhibitor of ex vivo myometrial contractions than indomethacin and aminophylline, but less potent than nifedipine and rolipram, making glycyl-H-1152 the third most potent contraction blocker assessed (Table 3). These data provide us with greater insight into the contraction blocking potency of these drugs, with glycyl-H-1152 in particular emerging as a potential novel tocolytic due to its substantial potency. Glycyl-H-1152 may be an excellent candidate for encapsulation into uterine-targeted nanoliposomes [53] or vaginally administered mucus penetrating nanoparticles [75] as novel tocolytic strategies for preventing preterm birth. Such platforms may also facilitate the administration of hydrophobic drugs, such as HC-067047. Further studies are warranted to assess the tocolytic efficacy and safety of these agents in vivo using preterm birth models.

## Supplementary Information

Below is the link to the electronic supplementary material.

Supplementary file2 Figure S1. Longevity of spontaneous myometrial contractions ex vivo. (A) A representative trace showing spontaneous pregnant human myometrial contractions recorded ex vivo for > 7 h (n = 5). Panels (B), (C), (D) and (E) show the mean contraction resting tension, amplitude, frequency, and AUC calculated during each 1 h period (expressed as a percentage of baseline contractility). There was no significant change in resting tension, amplitude, frequency, or AUC over the > 7 h recording period. Comparisons were made between baseline and the individual 60 min periods using ordinary one-way ANOVA followed by Dunnett’s multiple comparisons test. A probability (P) value of < 0.05 was considered as statistically significant (JPG 3.35 MB)

Supplementary file1 Figure S2. Assessment of contraction parameters. Illustration showing how contraction amplitude and frequency were measured and AUC determined. The horizontal blue dotted line indicates the baseline that was used as lower border for calculating AUC (JPG 976 KB)

Supplementary file3 Figure S3. Representative traces showing the effect of cumulative doses of drug vehicles on contractions. Spontaneously contracting pregnant human myometrial strips were treated with cumulative doses (10 μL each) of (A) DMSO(0, 0.06, 0.12, 0.18, 0,24, 0.30, 0.36, 0.42% v/v), (B) KREBS buffer (0, 0.06, 0.12, 0.18, 0,24, 0.30, 0.36, 0.42% v/v) and (C) Milli-Q water(0, 0.06, 0.12, 0.18, 0,24, 0.30% v/v). Dotted lines indicate the points at which the treatment was added to the bath(JPG 3723 KB)

## Data Availability

Not applicable.
