# Supplementary figures and images for: Assessing the Potency of the Novel Tocolytics 2-APB, Glycyl-H-1152, and HC-067047 in Pregnant Human Myometrium
Source: Reprod Sci. 2022 Jun 17;30(1):203–20. doi: 10.1007/s43032-022-01000-2 (PMC9810572; doi:10.1007/s43032-022-01000-2)

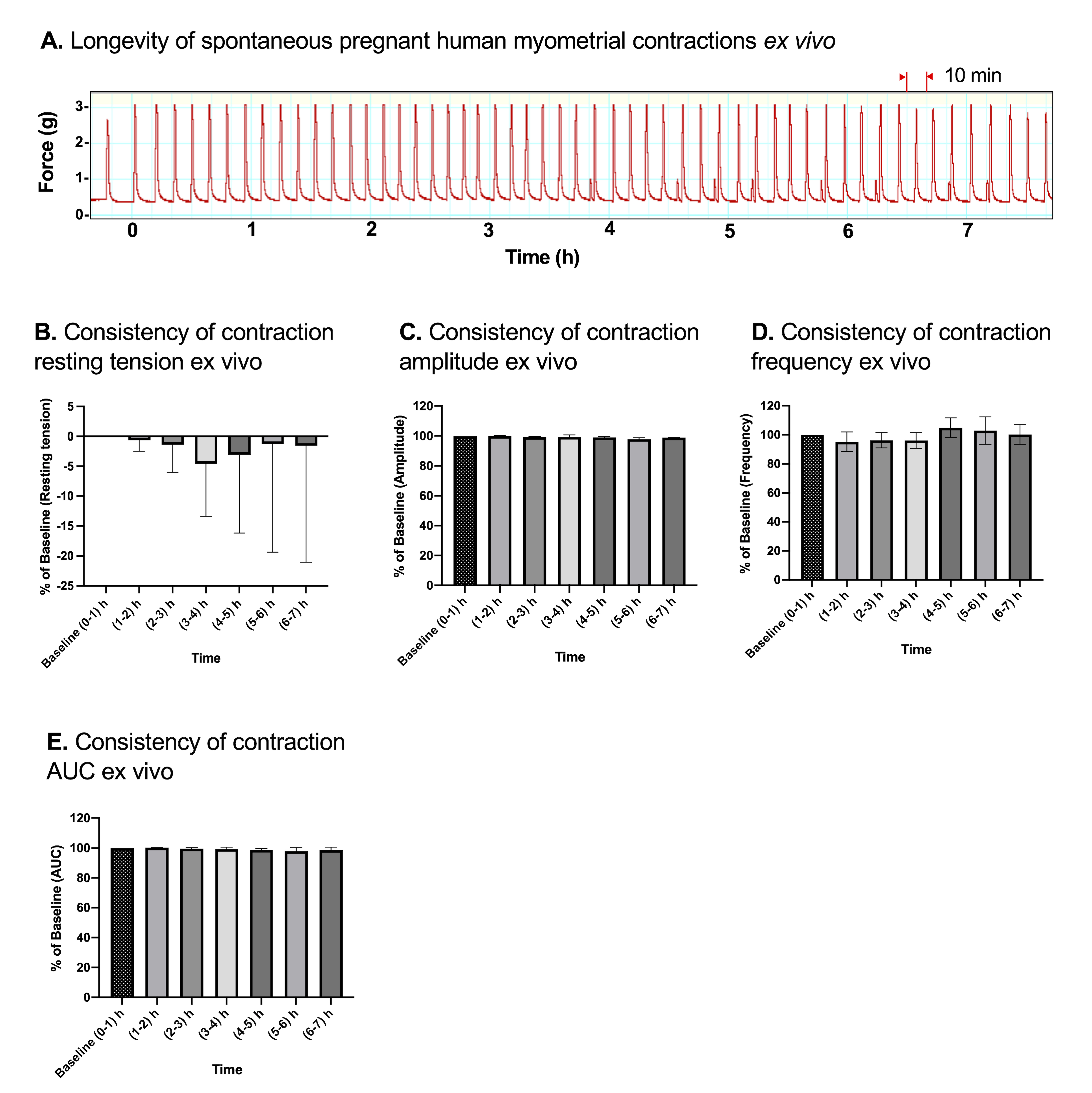

Supplement: Supplementary file 1 — Supplementary file2 Figure S1. Longevity of spontaneous myometrial contractions ex vivo. (A) A representative trace showing spontaneous pregnant human myometrial contractions recorded ex vivo for > 7 h (n = 5). Panels (B), (C), (D) and (E) show the mean contraction resting tension, amplitude, frequency, and AUC calculated during each 1 h period (expressed as a percentage of baseline contractility). There was no significant change in resting tension, amplitude, frequency, or AUC over the > 7 h recording period. Comparisons were made between baseline and the individual 60 min periods using ordinary one-way ANOVA followed by Dunnett’s multiple comparisons test. A probability (P) value of < 0.05 was considered as statistically significant (JPG 3.35 MB) [file 43032_2022_1000_MOESM1_ESM.jpg]

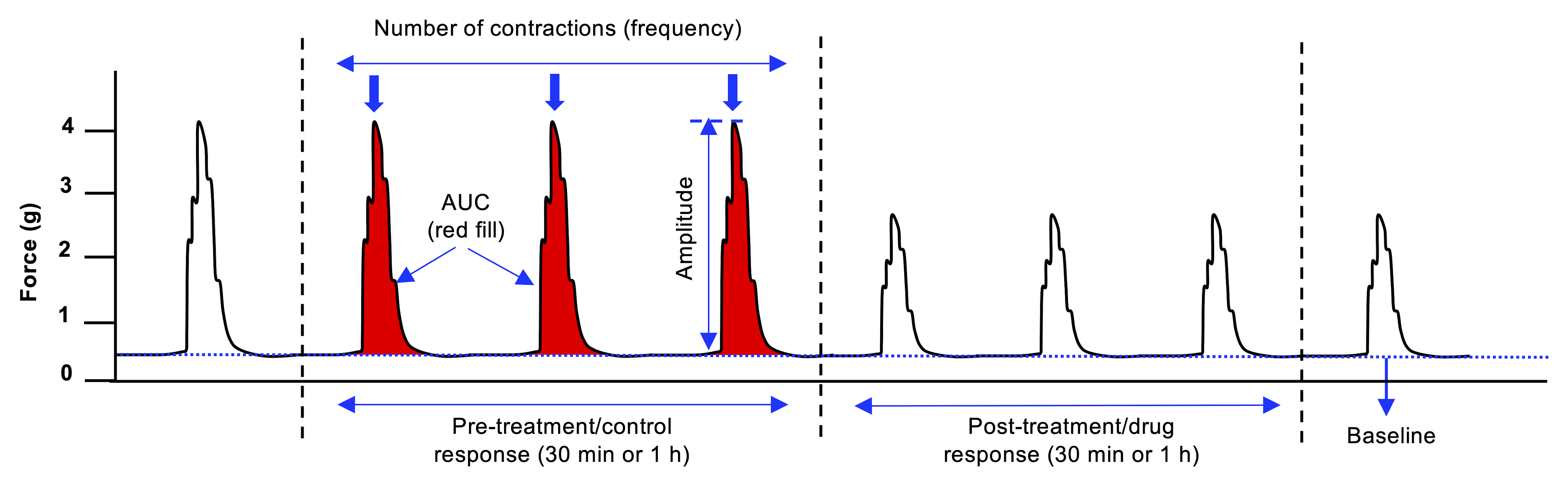

Supplement: Supplementary file 2 — Supplementary file1 Figure S2. Assessment of contraction parameters. Illustration showing how contraction amplitude and frequency were measured and AUC determined. The horizontal blue dotted line indicates the baseline that was used as lower border for calculating AUC (JPG 976 KB) [file 43032_2022_1000_MOESM2_ESM.jpg]

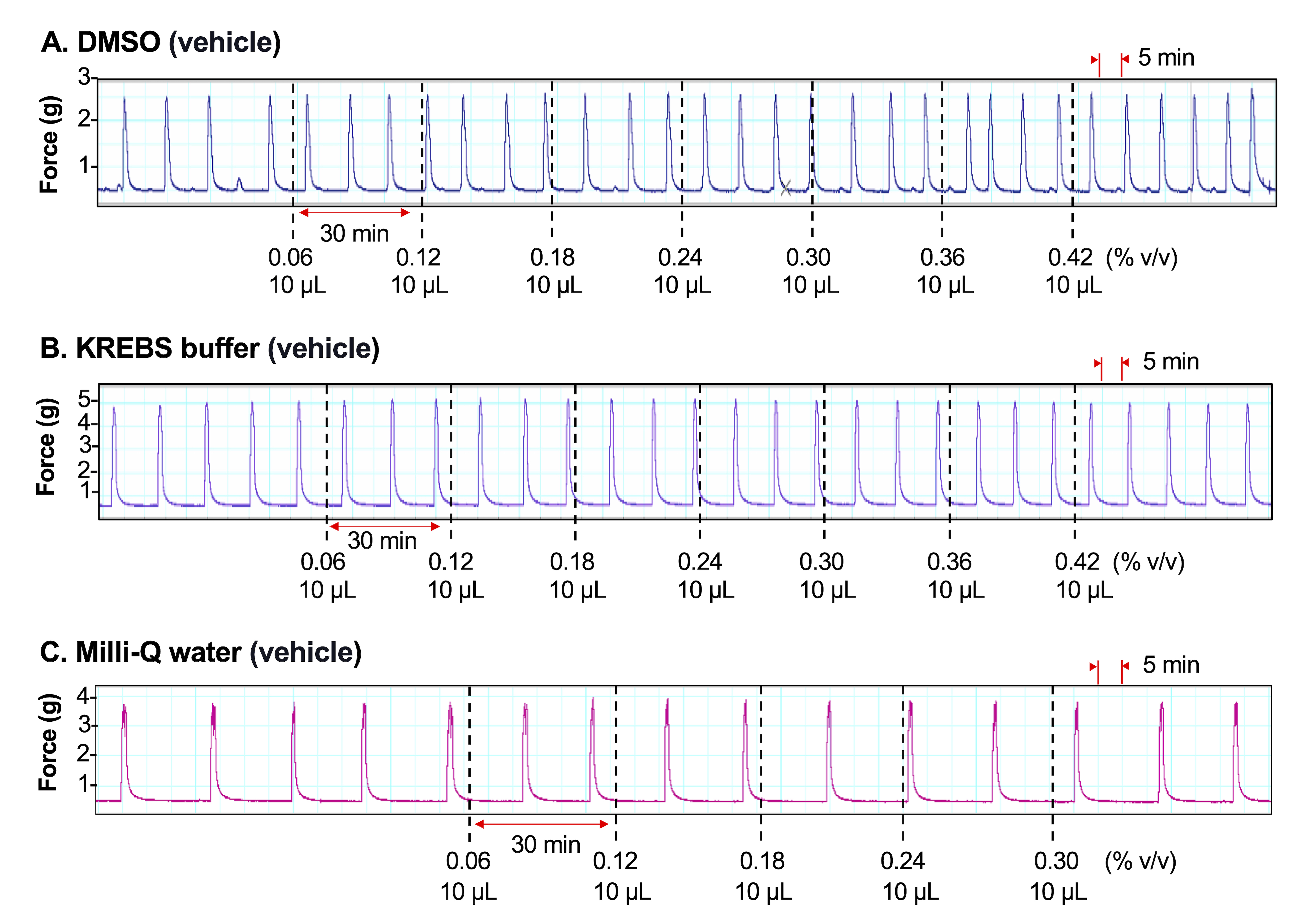

Supplement: Supplementary file 3 — Supplementary file3 Figure S3. Representative traces showing the effect of cumulative doses of drug vehicles on contractions. Spontaneously contracting pregnant human myometrial strips were treated with cumulative doses (10 μL each) of (A) DMSO(0, 0.06, 0.12, 0.18, 0,24, 0.30, 0.36, 0.42% v/v), (B) KREBS buffer (0, 0.06, 0.12, 0.18, 0,24, 0.30, 0.36, 0.42% v/v) and (C) Milli-Q water(0, 0.06, 0.12, 0.18, 0,24, 0.30% v/v). Dotted lines indicate the points at which the treatment was added to the bath(JPG 3723 KB) [file 43032_2022_1000_MOESM3_ESM.jpg]
